# Supplementary material for: Kallikrein-11, in Association with Coiled-Coil Domain Containing 25, as a Potential Prognostic Marker for Cholangiocarcinoma with Lymph Node Metastasis
Source: Molecules. 2021 May 22;26(11):3105. doi: 10.3390/molecules26113105 (PMC8196963; doi:10.3390/molecules26113105)
Supplement: Supplementary file 1 [file molecules-26-03105-s001.zip › molecules-1166888-supplementary.pdf]

Supplementary Material

Table S1. The characterization of CCDC25-binding protein profiling

Gene name	Protein name	Protein class	Protein location	Protein intensity
KLK11	Kallikrein-11	Serine protease	Intracellular, Secreted (different isoforms)	3860700
FAM105A	Family with sequence similarity 105, member A	Enzymes	Membrane	1904500
PIP5K1C	Phosphatidylinositol 4-phosphate 5-kinase type-1 gamma	Kinase activator	Intracellular	770560
